# Temporal trends in depression-related hospitalizations and suicide among patients with inflammatory bowel disease

**DOI:** 10.1093/jcag/gwag015

**Published:** 2026-05-14

**Authors:** Ante Markovinović, Christopher Ma, Abdel-Aziz Shaheen, Stephanie Coward, Gilaad G Kaplan

**Affiliations:** Department of Medicine, University of Calgary, Calgary, AB T2N 4Z6, Canada; Department of Community Health Sciences, University of Calgary, Calgary, AB T2N 4Z6, Canada; Department of Medicine, University of Calgary, Calgary, AB T2N 4Z6, Canada; Department of Community Health Sciences, University of Calgary, Calgary, AB T2N 4Z6, Canada; Department of Medicine, University of Calgary, Calgary, AB T2N 4Z6, Canada; Department of Community Health Sciences, University of Calgary, Calgary, AB T2N 4Z6, Canada; Department of Medicine, University of Calgary, Calgary, AB T2N 4Z6, Canada; Department of Community Health Sciences, University of Calgary, Calgary, AB T2N 4Z6, Canada; Department of Medicine, University of Calgary, Calgary, AB T2N 4Z6, Canada; Department of Community Health Sciences, University of Calgary, Calgary, AB T2N 4Z6, Canada

**Keywords:** mental health, Crohn’s disease, ulcerative colitis, hospitalization, epidemiology

## Abstract

**Background:**

Individuals with inflammatory bowel disease (IBD) are at higher risk of depression. However, data on depression-related hospitalizations and suicide death remain limited.

**Methods:**

This population-based study used administrative data to identify annual prevalent cohorts of individuals with IBD and matched controls between fiscal years 2005/2006 to 2021/2022. The primary outcomes were hospitalization for depression and suicide death. An IBD incident cohort was used to estimate the 1-, 3-, and 5-year cumulative incidence with 95% confidence intervals (CIs). Temporal trends were evaluated by calculating the average annual percent change (AAPC) using Poisson or negative binomial regression models. Conditional logistic regression estimated the odds ratio (OR) for depression-related hospitalizations between individuals with and without IBD.

**Results:**

Individuals with IBD had higher odds of depression-related hospitalization than matched controls (OR = 1.52; 95% CI, 1.38, 1.67). Over time, depression-related hospitalizations decreased in those with IBD (AAPC: −3.38%; 95% CI, −4.72, −2.03), driven by Crohn’s disease (AAPC: −4.76; 95% CI, −7.06, −2.39), whereas rates were stable in ulcerative colitis (AAPC: −1.71%; 95% CI, −4.46, 1.11). Hospitalization rates for depression in IBD declined in adults but were stable for pediatric patients (2.56%; 95% CI, −7.17, 13.30). Suicide deaths occurred in 0.21% (95% CI, 0.17, 0.25) of those with IBD and were stable over time (AAPC: −0.99%; 95% CI, −2.44, 0.48). The 1-, 3-, and 5-year cumulative incidence of depression-related hospitalization in the IBD incident cohort was 0.16% (95% CI, 0.12, 0.22), 0.41% (95% CI, 0.33, 0.50), and 0.63% (95% CI, 0.52, 0.75), respectively.

**Conclusions:**

Depression-related hospitalizations declined among individuals with IBD, particularly those with Crohn’s disease, whereas suicide rates were stable.

## Introduction

Inflammatory bowel disease (IBD) is a chronic inflammatory condition of the gastrointestinal tract comprising Crohn’s disease and ulcerative colitis, which affects more than 5 million persons globally.^1^^,^^2^ Currently, the prevalence of IBD in Canada is approximately 0.8% and is forecasted to rise to 1% by 2035.^3^^,^^4^ In addition to debilitating physical manifestations such as abdominal pain, bloody diarrhea, and fecal urgency, individuals with IBD are at significantly increased risk of mental health disorders, such as depression, even after achieving objectively defined remission from a bowel perspective.^5^^,^^6^

Notably, depression may occur either before or after the onset of IBD. This susceptibility to depression is partly due to the chronic, episodic nature of IBD that often demands major shifts in coping strategies and mindset, as research has shown that those who struggle to adjust to their new diagnosis of IBD were more likely to be depressed.^7^ Furthermore, research has established the relationship between depression and IBD to be bidirectional, whereby depression is associated with an increased risk of developing IBD, while active disease flares are associated with heightened depressive symptomology.^8^^,^^9^

This relationship is also affected by medications used to treat IBD, such as corticosteroids, which can impact an individual’s psychological wellbeing. Studies have found that those taking oral prednisone were more likely to report feeling depressed.^10^^,^^11^ Given this compounding relationship, some individuals with IBD face an elevated risk of severe depressive symptomology requiring hospitalization and, in extreme cases, suicidality.^12^

The literature on extreme outcomes of depression in patients with IBD, including hospitalization for depression and suicide, is limited. This highlights a crucial gap in determining the burden of depression and suicide in those with IBD. Consequently, this study aimed to calculate the 1-, 3-, and 5-year incidence risk of depression-related hospitalizations and to assess temporal trends of depression-related hospitalization and suicide in individuals with IBD.

## Methods

This population-based study used administrative data from Alberta, Canada to identify individuals with IBD using a validated algorithm applied across the full observation period. Annual point prevalence cohorts of individuals with IBD were derived for each fiscal year (FY) (ie, April 1 to March 31 [eg, FY 2005 = April 1, 2005, to March 31, 2006]) 2005/2006 to 2021/2022. Within each FY, outcomes assessed included: (1) depression-related hospitalization; and (2) suicide death. The administrative healthcare data used includes the following databases: Discharge Abstract Database (DAD), (inpatient hospitalizations); National Ambulatory Care Reporting System/Alberta Ambulatory Care Reporting System (NACRS/AACRS), outpatient and same-day procedures; Practitioner Claims, fee-for-service billing interactions; Vital Statistics, identifies deaths; and Provincial Registry, identifies whether an individual is a resident of Alberta in a given year.^13^

### Study population

Identification of the IBD prevalent cohort from healthcare-based administrative data requires the use of DAD, NACRS/AACRS, and Practitioner Claims. Individuals are identified using IBD International Classification of Disease (ICD) codes (ICD-9: 555.X, 556.X; ICD-10-CA: K50.X, K51.X). An Alberta-specific validated algorithm was applied, defining a prevalent case of IBD as those with IBD-related ICD codes in: (1) 2 DAD admissions; (2) 4 Practitioner Claims; or (3) 2 AACRS/NACRS admissions within 2 years.^14^ This algorithm has a sensitivity of 78.0% and a specificity of 99.8%.^14^ Individuals were identified as being Alberta residents, or having left, with Provincial Registry data—this dataset captures individuals who are on the Alberta Health Care Insurance Plan (AHCIP), which captures approximately 99% of the Alberta population.^15^ As IBD is a chronic disease, once an individual meets AHCIP criteria, they are included in all subsequent years as a prevalent case and only removed when their AHCIP coverage ends (eg, migration from the province or death).^15^

**Figure 1:**
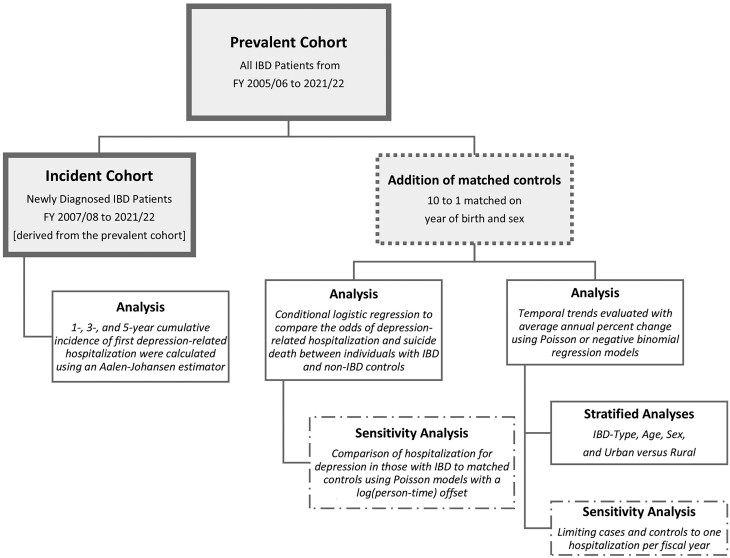
Flowchart of study design, cohort derivation, and associated analyses.

An algorithm using administrative data for identification of Crohn’s disease, ulcerative colitis, and IBD unclassified has previously been validated, which detects Crohn’s disease with a sensitivity of 93.5% and specificity of >99%, while detecting ulcerative colitis with a sensitivity of 86.3% and a specificity of >99%.^14^ The resulting prevalent IBD cohort spans FY2005/2006 to FY2021/2022. Study period started at the first IBD-related health care encounter recorded in the administrative data for each individual. Prevalent IBD cases were 10 to 1 age and sex matched to non-IBD controls. All individuals in the Provincial Registry during the study period, who were not previously identified as IBD cases, comprised the pool of eligible controls. A macro was applied to randomly select 10 controls per case, matched on age based on year of birth and sex. Cohort derivation and associated analyses are summarized in Figure 1.

### Incident cohort

A washout period was applied to identify incident cases of IBD (adult (≥18 years): 5-year washout; pediatric (<18 years): 3-year washout).^16^ The incident cohort spanned FY 2007/2008 to 2021/2022.

### Outcomes

Outcomes of interest were: (1) hospitalization for depression; and (2) suicide death. A case of hospitalization due to depression was defined as an individual having a primary diagnostic code for depression-related inpatient admission ICD code (ICD-10-CA: F32.X, F33.0-3, F33.8-9, F34.1 F41.2).^17^^,^^18^ Suicide deaths were ascertained using the Vital Statistics registry, which captures both in-hospital and out-of-hospital deaths, using ICD-10-CA codes X60-84 and Y87.^19^ These outcomes were assessed annually from FY 2005/2006 to 2021/2022, with individuals censored at migration out of province, death, or the end of the study period.

### Covariates

The covariates of interest included: age (pediatrics [<18 years of age], adults [≥18-59], and elderly [≥60]), sex (male and female), IBD type (CD and UC + IBD Unclassified [IBDU]), and geography (rural and urban based on postal code).^20^ If an individual did not have a rural or urban designation for a given year then the matching year’s admissions were dropped from the geography analysis.

### Statistical analysis

Statistical analyses were performed using Stata v.19.0 (StataCorp, College Station, TX, United States).^21^ For both IBD cases and controls, the total number of individuals with depression-related hospitalizations and suicide death (overall and stratified by: age, sex, and geography) were calculated. Age was based on first hospitalization for depression and date of death for suicide. IBD cases were stratified by IBD type. The number of admissions per individual (1, 2 to 3, and >3) were calculated for individuals who were hospitalized for depression. The proportion of individuals with depression-related hospitalizations (one hospitalization per individual) or suicide deaths were calculated based number of individuals in the IBD and control cohorts, respectively. Chi-squared tests compared the proportions between IBD cases and controls and a 2-sample t-test was used to compare mean ages (with standard deviation). The odds ratios (OR) and corresponding 95% confidence intervals (CI) were calculated with conditional logistic regression to compare the odds of depression-related hospitalization and suicide death between individuals with IBD and non-IBD controls, with each individual counted only once. The model was adjusted for socioeconomic status (SES) by including a 5-level social deprivation index as a categorical covariate.

The primary analysis used the overall prevalent cohort to calculate annual rates of depression-related hospitalizations per 100 000 person-years. The average annual percent change (AAPC) and corresponding 95% CIs were estimated using Poisson or negative binomial regression models, depending on data dispersion. AAPCs quantify temporal trends in depression-related hospitalization rates; CIs including 0% indicate no statistically significant change in the trend. AAPCs were stratified by age, sex, and geographic location in both cases and controls, with cases also stratified by IBD type. A likelihood ratio test assessed whether AAPCs differed significantly within the covariate stratifications and between IBD cases and controls. The overall AAPCs, with 95% CI, for individuals with IBD and non-IBD controls were calculated for suicide deaths using log-binomial regression.

In a secondary analysis, the IBD incident cohort was used to calculate the 1-, 3-, and 5-year cumulative incidence (%), with 95% CI, of first depression-related hospitalization using an Aalen-Johansen estimator that adjusts for the competing risk of death and handles right censoring. Follow-up times were calculated from the diagnosis date of each incident IBD case. For participants with multiple admissions, only the first qualifying hospitalization within the follow-up period was considered eligible.

### Sensitivity analyses

A sensitivity analysis was conducted to determine the AAPC of the proportion of individuals who have a depression-related hospitalization each year (2005/2006 to 2021/2022); limiting cases and controls to one hospitalization per FY. In a sensitivity analysis, we compared hospitalization for depression in those IBD to matched controls using Poisson models with a log(person-time) offset. This method accounts for differing follow-up by censoring at death, emigration, or study end, reporting incidence rate ratios (IRRs) with 95% CI adjusted for SES.

## Results

Between FY 2005/2006 and 2021/2022, there were 869 total depression-related hospitalizations among individuals with IBD and 5016 hospitalizations in controls. These hospitalizations occurred in 550 individuals with IBD (1.29%; 95% CI, 1.19, 1.40) and 3389 controls (0.85%; 95% CI, 0.82, 0.88) (Table 1).

**Table 1:** Case and control demographics for depression hospitalizations.

	IBD cases (*n* = 550)	Controls (*n* = 3389)	Comparison between cases and controls (*P*-values)
**Total admissions, *n***	869	5016	N/A
**Number of admissions, *n (*%)**			0.774
** 1 admission**	403 (73.3)	2529 (74.6)	
** 2 to 3 admissions**	118 (21.5)	683 (20.2)	
** >3 admissions**	29 (5.2)	177 (5.2)	
**Sex, *n (*%)**			0.305
** Female**	330 (60.0%)	2111 (62.3%)	
** Male**	220 (40.0%)	1278 (37.7%)	
**Mean age at first admission (SD)**	48.64 (16.31)	47.67 (18.60)	0.249
**Age at first admission, *n* (%)**			0.002^a^
** <18**	11 (2.0%)	182 (5.4%)	
** 18-59**	401 (72.9%)	2314 (68.3%)	
** 60+**	138 (25.1%)	893 (26.3%)	
**IBD type, *n* (%)**			N/A
**Crohn’s disease**	326 (59.3%)	–	
**Ulcerative Colitis and IBDU**	224 (40.7%)	–	
**Geography, *n* (%)**			0.929
** Rural**	194 (35.3%)	1186 (35.0%)	
** Urban**	356 (64.7%)	2203 (65.0%)	

a Denotes statistical significance. Abbreviations: SD, standard deviation; IBDU, Inflammatory Bowel Disease-Unclassifiable; N/A, Not Applicable.

A majority of both IBD cases and controls experienced only one admission for depression with 73.3%, and 74.6%, respectively. The mean age at first admission of those hospitalized with depression was 48.6 (SD: 16.3) years for IBD cases and 47.7 (SD: 18.6) in controls. There were no significant differences between IBD cases and controls for: sex (*P* = .305), mean age at first admission (*P* = .249), and geography (*P* = .929). There was a significant difference in the stratification of age groups between IBD cases and controls (*P *= .002).

IBD cases were at a significantly increased odds of hospitalization for depression than controls (OR: 1.52; 95% CI, 1.38, 1.67). This relationship was consistent with a sensitivity analysis using a person-time analysis with an IRR was 2.03 (95% CI, 1.89-2.20).

From FY 2005/2006 to 2021/2022, there were 89 (0.21%; 95% CI, 0.17, 0.25) suicide deaths in IBD cases and 746 (0.19%; 95% CI, 0.17, 0.2) in controls (Table 2). Suicide deaths did not differ significantly between individuals with and without IBD (OR = 1.13; 95% CI, 0.91, 1.41). Additionally, there were no significant differences between IBD cases and controls for: age groups, sex, or geography (Table 2).

**Table 2:** Case and control demographics for suicide death.

	IBD cases	Controls	Comparison (*P*-value)
**Total, *n***	89	746	
**Sex, *n (*%)**			0.910
** Female**	27 (30.3%)	222 (29.8%)	
** Male**	62 (69.7%)	524 (70.2%)	
**Mean age (SD)**	49.31 (14.47)	49.43 (15.73)	0.948
**Age, *n* (%)**			0.718
** <59^a^**	64 (71.9%)	547 (73.4%)	
** 60+**	25 (28.1%)	199 (26.7%)	
**IBD type, *n* (%)**		N/A	N/A
** Crohn’s disease**	57 (64.0%)		
** Ulcerative Colitis & IBDU**	32 (36.0%)		
**Geography, *n* (%)**			0.668
** Rural**	25 (28.1%)	226 (30.3%)	
** Urban**	64 (71.9%)	520 (69.7%)	

a Age band <18 and 18-59 collapsed due to small cell size (<10) in pediatric age group in controls. No <18 in cases. Abbreviations: SD, standard deviation; IBDU, Inflammatory Bowel Disease-Unclassifiable; N/A, Not Applicable.

The 1-, 3-, and 5-year cumulative incidence of depression-related hospitalization in incident cases of IBD, accounting for competing risks, was 0.16% (95% CI, 0.12, 0.22), 0.41% (95% CI, 0.33, 0.50), and 0.63% (0.52, 0.75), respectively.

**Figure 2:**
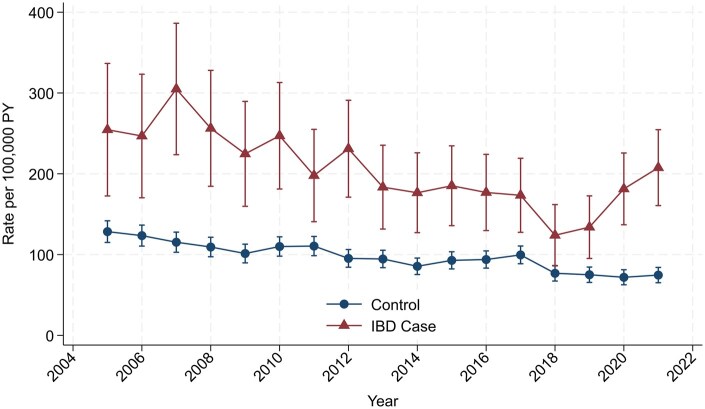
Historical trends in depression hospitalization in IBD cases and matched controls from 2005/2006 to 2021/2022. Hospitalization rates for depression are reported as cases per 100 000 person-years with 95% confidence intervals.

Hospitalizations for depression, from FY 2005/2006 to 2021/2022, significantly decreased in both IBD cases (−3.38%; 95% CI, −4.72, −2.03) and controls (−3.22%; 95% CI, −3.77, −2.66), and were not statistically different (*P *= .821) (Figure 2, Table 3).

**Table 3:** AAPCs for depression hospitalization in IBD cases and controls from 2005/2006 to 2021/2022.

	Cases		Controls		Comparison of AAPCs for stratum between cases and controls (*P*-value)
	**AAPC (95% CI)**	**Comparison between stratum (*P*-value)**	**AAPC (95% CI)**	**Comparison between stratum (*P*-value)**	
**CD**	−4.76^a^ (−7.06, −2.39)	0.015^a^	N/A	N/A	N/A
**UC**	−1.71 (−4.46, 1.11)				N/A
**IBD/overall**	−3.38^a^ (−4.72, −2.03)	N/A	−3.22^a^ (−3.77, −2.66)		0.821
**Pediatrics (<18)**	2.56 (−7.17, 13.30)	0.002^a^	5.94^a^ (3.05, 8.90)	<0.001^a^	0.543
**Adults (18-59)**	−2.10^a^ (−4.06, −0.10)		−3.46^a^ (−4.13, −2.78)		0.123
**Elderly (60+)**	−7.20^a^ (−9.62, −4.70)		−4.43^a^ (−5.46, −3.39)		0.045^a^
**Female**	−2.93^a^ (−4.62, −1.21)	0.446	−4.22^a^ (−5.07, −3.37)	<0.001^a^	0.170
**Male**	−4.00^a^ (−6.15, −1.81)		−1.42^a^ (−2.34, −0.48)		0.034^a^
**Rural**	−6.38^a^ (−8.60, −4.11)	0.001^a^	−4.20^a^ (−5.11, −3.28)	0.003^a^	0.080
**Urban**	−1.72^a^ (−3.40, −0.01)		−2.44^a^ (−3.13, −1.74)		0.438

a Denotes statistical significance.

Abbreviations: AAPC, average annual percentage change; CD, Crohn’s disease; CI, confidence interval; IBD, inflammatory bowel disease; UC, ulcerative colitis.

Hospitalization rates for depression decreased in those with Crohn’s disease (AAPC: −4.76%; 95% CI, −7.06, −2.39) and remained stable in those with ulcerative colitis (AAPC: −1.71%; 95% CI, −4.46, 1.11) (Table 3, Figure S1). Hospitalization rates for depression significantly decreased among females (AAPC: −2.93%; 95% CI, −4.62, −1.21) and males (AAPC: −4.00%; 95% CI, −6.15, −1.81) with IBD (Table 3, Figure S2). Depression-related hospitalization rates declined among adults aged 18-59 years (−2.10%; 95% CI, −4.06 to −0.10) and older adults aged ≥60 years (−7.20%; 95% CI, −9.62 to −4.70), whereas rates remained stable in pediatric patients with IBD (2.56%; 95% CI, −7.17 to 13.30) (Table 3, Figure S3). Finally, among individuals with IBD, depression-related hospitalization rates significantly declined in both urban (−1.72%; 95% CI, −3.40 to −0.01) and rural areas (−6.38%; 95% CI, −8.60 to −4.11) (Table 3, Figure S4).

In a sensitivity analysis limiting cases and controls to one hospitalization per FY, the proportion of individuals with a depression-related hospitalization significantly declined in both IBD cases (−4.47%; 95% CI, −5.90 to −3.01) and controls (−2.90%; 95% CI, −3.49 to −2.31).

Suicide deaths in those with IBD and in controls remained stable, with AAPCs of −0.99% (95% CI, −2.44, 0.48) and −1.35% (95% CI, −5.58, 3.06), respectively. There was no significant difference in their AAPCs (*P* = .875).

## Discussion

This study represents the first population-based study to assess depression-related hospitalization and suicide in those with IBD, providing insight into the burden of severe mental health outcomes in a population highly susceptible to mood disorders such as depression. Depression is a major health concern with 0.63% of individuals with IBD hospitalized for depression within 5-years of diagnosis, and approximately 1 in 500 dying by suicide over the 17-year study period. Importantly, from FY 2005 to 2021, depression-related hospitalizations in those with IBD decreased by 3.4% each year, following similar trends observed in the control population. The decline in depression-related hospitalization rates was driven by Crohn’s disease, whereas rates in ulcerative colitis remained stable over the study period. While depression is a common comorbidity of IBD, these findings suggest a shift from hospital-based to ambulatory care for managing depression in the IBD population.

Depression affects approximately 15% of those with IBD.^5^ This study examines the most severe outcomes, hospitalization for depression or suicide after a diagnosis of IBD, as these represent the greatest mental health impacts on people with IBD and their caregivers.^22^ The cumulative incidence of depression-related hospitalization after IBD diagnosis increased by more than 0.1% per year. Research has consistently shown that individuals with IBD have a higher risk of mental health comorbidities than those without IBD.^23^ Similarly, we observed that the odds of depression-related hospitalization was significantly higher among individuals with IBD compared with their matched controls. Over the study period, 0.21% individuals with IBD died by suicide. In the context of the high prevalence of depression in outpatient settings, these findings support the importance of screening for depression and suicidal ideation in IBD patients and ensuring access to appropriate mental health resources, including psychological counseling and pharmacotherapy.^24^^,^^25^

Depression-related hospitalization rates declined at a similar pace in individuals with IBD and controls (approximately 3% per year). When stratified by IBD type, this decline was driven by Crohn’s disease (4.8% per year), while rates in ulcerative colitis remained stable. Although this study was not designed to identify the causes of these trends, improvements in IBD management over the past 2 decades may have contributed. The widespread adoption of more effective treatments, including tumor necrosis factor antagonists, vedolizumab, ustekinumab, Janus kinase inhibitors, and IL23-targeted agents, has improved disease control, reduced complications, and decreased the need for surgery.^26–28^ Improved outcomes may positively influence mental health by enhancing quality of life, alleviating IBD symptoms, and reducing systemic inflammation.^29^ These effects maybe more pronounced in Crohn’s disease, where patients have a greater need for surgery and are prone to postoperative recurrence. In contrast, patients with severe ulcerative colitis may achieve long-term disease control following proctocolectomy. These differences may explain the divergent temporal trends in depression-related hospitalizations observed between Crohn’s disease and ulcerative colitis.

The parallel decline in hospitalization rates observed in the control population suggests that broader, non-IBD-related and non-treatment factors also influenced hospitalization trends. The general decrease in stigma surrounding depression and increased awareness of mental health susceptibility in those with IBD may have contributed to lower hospitalization rates for depression.^30^ Over the past 2 decades, public attitude toward depression have evolved, with reduced judgment toward those suffering from depression and greater recognition of the biopsychosocial underpinnings of depression.^31^ Additionally, clinical awareness of mental health vulnerability in the IBD population has increased over recent years, leading to improved access to mental health resources.^5^ This increased recognition and support may have facilitated earlier intervention, potentially mitigating depressive symptoms and reducing the need for hospitalization.^23^

While hospitalization rates for depression decreased in adults, the pediatric-onset IBD population remained stable. This difference might be attributable to the increasing incidence of pediatric-onset IBD or the psychological immaturity of pediatrics with IBD, the latter of which may limit their capacity to cope with their disease and lead to more severe outcomes of depression.^32^^,^^33^

Expanded community-based interventions have likely improved outpatient treatment of depression, including urgent psychiatric services and crisis support programs designed to stabilize patients outside of hospital.^34^^,^^35^ For example, the Alberta Mental Health Line, a multidisciplinary line comprising psychiatric nurses, social workers, and psychologists, provides crisis support, triage, and mental health screening.^36^ Collectively, these expanded community-based resources have likely contributed to the observed decline in depression-related hospitalizations.

Despite system-wide reductions in depression-related hospitalization, suicide deaths in those with IBD and their matched controls remained stable across time suggesting that the clinical, societal, and healthcare system factors contributing to reduced hospitalizations have not effectively translated into suicide prevention. As a result, identifying and addressing suicide risk in the IBD population remains a critical gap. While healthcare providers are increasingly diagnosing and discussing depression in individuals with IBD, screening for suicidal ideation may still be lacking.

This study should be interpreted in the context of its limitations. We focused on depression-related hospitalization and suicide because they represent the most severe consequences of depression, for which population-level data are limited. However, these outcomes capture only the most severe end of the disease spectrum and do not reflect the broader burden of depression managed in outpatient settings and did not account for suicide attempts that did not result in death. Although coding of suicide is accurate, some misclassification is possible, particularly for substance-related deaths where intent may be difficult to establish.^19^ While misclassification is a common concern in administrative database research, the use of a validated algorithm to identify the IBD population helps mitigate this issue. Disease severity, which is not captured in administrative data, was not accounted for, nor were medications, which can impact the relationship between IBD and depression.^37^ Depression-related hospitalization was identified using a validated approach based on the primary diagnostic coding of the 25 available discharge diagnoses. As a result, some hospitalizations for depression may have been missed if depression was coded as a secondary diagnosis, and this approach did not did not capture individuals with IBD admitted primarily for IBD-related reasons who had concurrent depression. Finally, insufficient sample sizes did not permit the stratification of suicide data in the same manner as depression hospitalizations.

This population-based study found that hospitalizations for depression have decreased in those with Crohn’s disease, possibly due to clinical advancements in treating IBD, increasing societal recognition and acceptance of mental health disorders, and shifting healthcare systems. Depression hospitalizations did not decline in pediatric IBD patients, who may be vulnerable to depression outcomes given their psychological immaturity and inability to cope with IBD. Despite decreasing trends in depression hospitalization, suicide deaths in those with IBD remained stable during the study period, indicating that advancements in recognizing and treating depression have not translated to this outcome. Considering this, gastroenterologists and primary care physicians should prioritize increased screening for depression and suicidal ideation in those with IBD. Future research should investigate the prevalence of suicidal ideation in individuals with IBD and assess whether early psychological intervention can help reduce suicide risk.

## Supplementary Material

gwag015_Supplementary_Data

## Data Availability

To comply with Alberta’s Health Information Act, the dataset cannot be made publicly available. The data used in this study are held securely in de-identified form on a protected server at the University of Calgary and were provided by the Alberta Strategy for Patient-Oriented Research Support Unit (AbSPORU). Legal data-sharing agreements between the researchers, AbSPORU, and the data providers (eg, healthcare organizations and government) prohibit public release of the dataset.
