# Supplementary material for: Temporal trends in depression-related hospitalizations and suicide among patients with inflammatory bowel disease
Source: J Can Assoc Gastroenterol. 2026 May 14;9(4):239–46. doi: 10.1093/jcag/gwag015 (PMC13436818; doi:10.1093/jcag/gwag015)
Supplement: Supplementary file 1 [file gwag015_supplementary_data.zip › DEPRESSION_SUPPLEMENT (10MAR2026).docx]

**Supplemental Figure 1.** *Historical trends in depression hospitalization by IBD type*

**Supplemental Figure 2.** *Historical trends in depression hospitalization by sex*

**Supplemental Figure 3.** *Historical trends in depression hospitalization by age*

**Supplementary Figure 4.** *Historical trends in depression hospitalization by urban/rural status*
