# Supplementary material for: Temporal trends in depression-related hospitalizations and suicide among patients with inflammatory bowel disease
Source: J Can Assoc Gastroenterol. 2026 May 14;9(4):239–46. doi: 10.1093/jcag/gwag015 (PMC13436818; doi:10.1093/jcag/gwag015)
Supplement: Supplementary file 1 [file gwag015_supplementary_data.zip › icmje-coi-form Combined March 30 2026.pdf]

ICMJE DISCLOSURE FORM

Date:
Your Name: Gilaad Kaplan
Manuscript Title: Temporal Trends in Depression-Related Hospitalizations and Suicide Among Patients with Inflammatory Bowel Disease
Manuscript number (if known): JCAG-2025-0083

In the interest of transparency, we ask you to disclose all relationships/activities/interests listed below that are related to the content of your manuscript. "Related" means any relation with for-profit or not-for-profit third parties whose interests may be affected by the content of the manuscript. Disclosure represents a commitment to transparency and does not necessarily indicate a bias. If you are in doubt about whether to list a relationship/activity/interest, it is preferable that you do so.

The following questions apply to the author's relationships/activities/interests as they relate to the current manuscript only.

The author's relationships/activities/interests should be defined broadly. For example, if your manuscript pertains to the epidemiology of hypertension, you should declare all relationships with manufacturers of antihypertensive medication, even if that medication is not mentioned in the manuscript.

In item #1 below, report all support for the work reported in this manuscript without time limit. For all other items, the time frame for disclosure is the past 36 months.

		**Name all entities with whom you have this relationship or indicate none (add rows as needed)**	**Specifications/Comments (e.g., if payments were made to you or to your institution)**
**Time frame: Since the initial planning of the work**			
1	All support for the present manuscript (e.g., funding, provision of study materials, medical writing, article processing charges, etc.) **No time limit for this item.**	____None	
**Time frame: past 36 months**			
2	Grants or contracts from any entity (if not indicated in item #1 above).	Ferring	Paid to institution
3	Royalties or licenses	____None	
4	Consulting fees	____None	

5	Payment or honoraria for lectures, presentations, speakers bureaus, manuscript writing or educational events	Honoraria for speaking: AbbVie, Pfizer, Jannssen, Takeda	Paid to me
		Educational Grants: AbbVie, Celltrion, Ferring, Janssen, Pfizer, Takeda.	Paid to the institution
6	Payment for expert testimony	____None	
7	Support for attending meetings and/or travel	____None	
8	Patents planned, issued or pending	TREATMENT OF INFLAMMATORY DISORDERS, AUTOIMMUNE DISEASE, AND PBC. UTI Limited Partnership, assignee. Patent WO2019046959A1. PCT/CA2018/051098. 7 Sept. 2018.	Patents are not on the market. Institutes and inventors (via the institutes) will receive money when they are licensed and if they reach the market.
9	Participation on a Data Safety Monitoring Board or Advisory Board	____None	
10	Leadership or fiduciary role in other board, society, committee or advocacy group, paid or unpaid	____None	
11	Stock or stock options	____None	
12	Receipt of equipment, materials, drugs, medical writing, gifts or other services	____None	
13	Other financial or non-financial interests	____None	

Please place an "X" next to the following statement to indicate your agreement:

__X_ I certify that I have answered every question and have not altered the wording of any of the questions on this form.

ICMJE DISCLOSURE FORM

**Date:**	3/27/2026
**Your Name:**	Ante Markovinović
**Manuscript Title:**	Temporal Trends in Depression-Related Hospitalizations and Suicide Among Patients with Inflammatory Bowel Disease
**Manuscript Number (if known):**	JCAG-2025-0083

In the interest of transparency, we ask you to disclose all relationships/activities/interests listed belo that are related to the content of your manuscript. "Related" means any relation with for-profit or no for-profit third parties whose interests may be affected by the content of the manuscript. Disclosure represents a commitment to transparency and does not necessarily indicate a bias. If you are in doub about whether to list a relationship/activity/interest, it is preferable that you do so.

The author's relationships/activities/interests should be defined broadly. For example, if your manuscript pertains to the epidemiology of hypertension, you should declare all relationships with manufacturers of antihypertensive medication, even if that medication is not mentioned in the manuscript.

In item #1 below, report all support for the work reported in this manuscript without time limit. For all other items, the time frame for disclosure is the past 36 months.

		**Name all entities with whom you have this relationship or indicate none (add rows as needed)**	**Specifications/Comments (e.g., if payments were made to you or to your institution)**
**Time frame: Since the initial planning of the work**			
**1**	All support for the present manuscript (e.g., funding, provision of study materials, medical writing, article	☒ **None**	
			Click the tab key to add additional rows

		Name all entities with whom you have this relationship or indicate none (add rows as needed)	Specifications/Comments (e.g., if payments were made to you or to your institution)
	processing charges, etc.) **No time limit for this item.**		
**Time frame: past 36 months**			
**2**	Grants or contracts from any entity (if not indicated in item #1 above).	☒ **None**	
**3**	Royalties or licenses	☒ **None**	
**4**	Consulting fees	☒ **None**	
**5**	Payment or honoraria for lectures, presentations, speakers bureaus,	☒ **None**	

		Name all entities with whom you have this relationship or indicate none (add rows as needed)	Specifications/Comments (e.g., if payments were made to you or to your institution)
	manuscript writing or educational events		
6	Payment for expert testimony	☒ None	
7	Support for attending meetings and/or travel	☒ None	
8	Patents planned, issued or pending	☒ None	
9	Participation on a Data Safety Monitoring Board or Advisory Board	☒ None	
10	Leadership or fiduciary role in other board, society,	☒ None	

		Name all entities with whom you have this relationship or indicate none (add rows as needed)	Specifications/Comments (e.g., if payments were made to you or to your institution)
	committee or advocacy group, paid or unpaid		
11	Stock or stock options	☒ None	
12	Receipt of equipment, materials, drugs, medical writing, gifts or other services	☒ None	
13	Other financial or non-financial interests	☒ None	

Please place an "X" next to the following statement to indicate your agreement:

☒ I certify that I have answered every question and have not altered the wording of any of the questions on this form.

ICMJE DISCLOSURE FORM

Date: 8/27/2021

Your Name: Christopher Ma

Manuscript Title: Temporal Trends in Depression-Related Hospitalizations and Suicide Among Patients with Inflammatory Bowel Disease

Manuscript Number (if known): JCAG-2025-0083

In the interest of transparency, we ask you to disclose all relationships/activities/interests listed belo that are related to the content of your manuscript. "Related" means any relation with for-profit or no for-profit third parties whose interests may be affected by the content of the manuscript. Disclosure represents a commitment to transparency and does not necessarily indicate a bias. If you are in doub about whether to list a relationship/activity/interest, it is preferable that you do so.

The author's relationships/activities/interests should be defined broadly. For example, if your manuscript pertains to the epidemiology of hypertension, you should declare all relationships with manufacturers of antihypertensive medication, even if that medication is not mentioned in the manuscript.

In item #1 below, report all support for the work reported in this manuscript without time limit. For all other items, the time frame for disclosure is the past 36 months.

		Name all entities with whom you have this relationship or indicate none (add rows as needed)	Specifications/Comments (e.g., if payments were made to you or to your institution)
		**Time frame: Since the initial planning of the work**	
**1**	All support for the present manuscript (e.g., funding, provision of study materials, medical writing, article	☒ **None**	
			Click the tab key to add additional rows

		Name all entities with whom you have this relationship or indicate none (add rows as needed)	Specifications/Comments (e.g., if payments were made to you or to your institution)
	processing charges, etc.) **No time limit for this item.**		
**Time frame: past 36 months**			
**2**	Grants or contracts from any entity (if not indicated in item #1 above).	☐ **None**	
		AbbVie, Eli Lilly, Ferring, Pfizer	
**3**	Royalties or licenses	☐ **None**	
		Springer Publishing	
**4**	Consulting fees	☐ **None**	
		AbbVie, Alimentiv, Amgen, Anaptys Bio, AVIR Pharma Inc, Bristol Myers Squibb, Celltrion, Domain Therapeutics, Eupraxia, Eli Lilly, Ferring, Forte Biosciences, Fresenius Kabi, Gilead, Janssen, McKesson, Merck, Mirador Therapeutics, Pendopharm, Pfizer, Roche, Sanofi, Takeda, Tillotts Pharma	
**5**	Payment or honoraria	☐ **None**	

		Name all entities with whom you have this relationship or indicate none (add rows as needed)	Specifications/Comments (e.g., if payments were made to you or to your institution)
	for lectures, presentations, speakers bureaus, manuscript writing or educational events	AbbVie, Amgen, AVIR Pharma Inc, Alimentiv, Bristol Myers Squibb, Eli Lilly, Ferring, Fresenius Kabi, Janssen, Merck, Organon, Pendopharm, Pfizer, Sanofi, Takeda, Tillotts Pharma	
6	Payment for expert testimony	☒ None	
7	Support for attending meetings and/or travel	☒ None	
8	Patents planned, issued or pending	☒ None	
9	Participation on a Data Safety Monitoring Board or Advisory Board	☐ None<br>Eupraxia, Forte Biosciences, Mage Bio	

		Name all entities with whom you have this relationship or indicate none (add rows as needed)	Specifications/Comments (e.g., if payments were made to you or to your institution)
10	Leadership or fiduciary role in other board, society, committee or advocacy group, paid or unpaid	☒ **None**	
11	Stock or stock options	☒ **None**	
12	Receipt of equipment, materials, drugs, medical writing, gifts or other services	☒ **None**	
13	Other financial or non-financial interests	☒ **None**	

Please place an "X" next to the following statement to indicate your agreement:

	Name all entities with whom you have this relationship or indicate none (add rows as needed)	Specifications/Comments (e.g., if payments were made to you or to your institution)
☒ I certify that I have answered every question and have not altered the wording of any of the questions on this form.		

ICMJE DISCLOSURE FORM

**Date:**	8/27/2021
**Your Name:**	Abdel-Aziz Shaheen
**Manuscript Title:**	Temporal Trends in Depression-Related Hospitalizations and Suicide Among Patients with Inflammatory Bowel Disease
**Manuscript Number (if known):**	JCAG-2025-0083

In the interest of transparency, we ask you to disclose all relationships/activities/interests listed belo that are related to the content of your manuscript. "Related" means any relation with for-profit or no for-profit third parties whose interests may be affected by the content of the manuscript. Disclosure represents a commitment to transparency and does not necessarily indicate a bias. If you are in doub about whether to list a relationship/activity/interest, it is preferable that you do so.

The author's relationships/activities/interests should be defined broadly. For example, if your manuscript pertains to the epidemiology of hypertension, you should declare all relationships with manufacturers of antihypertensive medication, even if that medication is not mentioned in the manuscript.

In item #1 below, report all support for the work reported in this manuscript without time limit. For all other items, the time frame for disclosure is the past 36 months.

		**Name all entities with whom you have this relationship or indicate none (add rows as needed)**	**Specifications/Comments (e.g., if payments were made to you or to your institution)**
**Time frame: Since the initial planning of the work**			
**1**	All support for the present manuscript (e.g., funding, provision of study materials, medical writing, article	☒ **None**	
			Click the tab key to add additional rows

		Name all entities with whom you have this relationship or indicate none (add rows as needed)	Specifications/Comments (e.g., if payments were made to you or to your institution)
	processing charges, etc.) **No time limit for this item.**		
**Time frame: past 36 months**			
**2**	Grants or contracts from any entity (if not indicated in item #1 above).	☒ **None**	
		Gilead, Novo Nordisk	
**3**	Royalties or licenses	☒ **None**	
**4**	Consulting fees	☐ **None**	
		Gilead, Ipsen, Novo Nordisk, BI	
**5**	Payment or honoraria for lectures, presentations, speakers bureaus,	☐ **None**	
		Novo Nordisk	

		Name all entities with whom you have this relationship or indicate none (add rows as needed)	Specifications/Comments (e.g., if payments were made to you or to your institution)
	manuscript writing or educational events		
6	Payment for expert testimony	☒ None	
7	Support for attending meetings and/or travel	☒ None	
8	Patents planned, issued or pending	☐ None	
		Patent WO2019046959A1. PCT/CA2018/051098. 7 Sept. 2018.	
9	Participation on a Data Safety Monitoring Board or Advisory Board	☒ None	
10	Leadership or fiduciary role in other board,	☒ None	

		Name all entities with whom you have this relationship or indicate none (add rows as needed)	Specifications/Comments (e.g., if payments were made to you or to your institution)
	society, committee or advocacy group, paid or unpaid		
11	Stock or stock options	☒ **None**	
12	Receipt of equipment, materials, drugs, medical writing, gifts or other services	☒ **None**	
13	Other financial or non-financial interests	☒ **None**	

Please place an "X" next to the following statement to indicate your agreement:

☒ I certify that I have answered every question and have not altered the wording of any of the questions on this form.

ICMJE DISCLOSURE FORM

**Date:**	8/27/2021
**Your Name:**	Stephanie Coward
**Manuscript Title:**	Temporal Trends in Depression-Related Hospitalizations and Suicide Among Patients with Inflammatory Bowel Disease
**Manuscript Number (if known):**	JCAG-2025-0083

In the interest of transparency, we ask you to disclose all relationships/activities/interests listed belo that are related to the content of your manuscript. "Related" means any relation with for-profit or no for-profit third parties whose interests may be affected by the content of the manuscript. Disclosure represents a commitment to transparency and does not necessarily indicate a bias. If you are in doub about whether to list a relationship/activity/interest, it is preferable that you do so.

The author's relationships/activities/interests should be defined broadly. For example, if your manuscript pertains to the epidemiology of hypertension, you should declare all relationships with manufacturers of antihypertensive medication, even if that medication is not mentioned in the manuscript.

In item #1 below, report all support for the work reported in this manuscript without time limit. For all other items, the time frame for disclosure is the past 36 months.

		Name all entities with whom you have this relationship or indicate none (add rows as needed)	Specifications/Comments (e.g., if payments were made to you or to your institution)
**Time frame: Since the initial planning of the work**			
**1**	All support for the present manuscript (e.g., funding, provision of study materials, medical writing, article	☒ **None**	
			Click the tab key to add additional rows

		Name all entities with whom you have this relationship or indicate none (add rows as needed)	Specifications/Comments (e.g., if payments were made to you or to your institution)
	processing charges, etc.) **No time limit for this item.**		
**Time frame: past 36 months**			
**2**	Grants or contracts from any entity (if not indicated in item #1 above).	☒ **None**	
**3**	Royalties or licenses	☒ **None**	
**4**	Consulting fees	☐ **None**	
		Sanofi	Paid to me
**5**	Payment or honoraria for lectures, presentations, speakers bureaus,	☒ **None**	

		Name all entities with whom you have this relationship or indicate none (add rows as needed)	Specifications/Comments (e.g., if payments were made to you or to your institution)
	manuscript writing or educational events		
6	Payment for expert testimony	☒ None	
7	Support for attending meetings and/or travel	☒ None	
8	Patents planned, issued or pending	☒ None	
9	Participation on a Data Safety Monitoring Board or Advisory Board	☒ None	
10	Leadership or fiduciary role in other board, society,	☒ None	

		Name all entities with whom you have this relationship or indicate none (add rows as needed)	Specifications/Comments (e.g., if payments were made to you or to your institution)
	committee or advocacy group, paid or unpaid		
11	Stock or stock options	☒ **None**	
12	Receipt of equipment, materials, drugs, medical writing, gifts or other services	☒ **None**	
13	Other financial or non-financial interests	☒ **None**	

Please place an "X" next to the following statement to indicate your agreement:

☒ I certify that I have answered every question and have not altered the wording of any of the questions on this form.
