# Supplementary material for: Temporal trends in depression-related hospitalizations and suicide among patients with inflammatory bowel disease
Source: J Can Assoc Gastroenterol. 2026 May 14;9(4):239–46. doi: 10.1093/jcag/gwag015 (PMC13436818; doi:10.1093/jcag/gwag015)
Supplement: Supplementary file 1 [file gwag015_supplementary_data.zip › Strobe Checklist.docx]

STROBE Statement—checklist of items that should be included in reports of observational studies

|  | Item No. | Recommendation | Page  No. | Relevant text from manuscript |
| --- | --- | --- | --- | --- |
| **Title and abstract** | 1 | (*a*) Indicate the study’s design with a commonly used term in the title or the abstract | 1 | Declining Depression Hospitalizations in Inflammatory Bowel Disease; Suicide Rates Remain Stable |
|  |  | (*b*) Provide in the abstract an informative and balanced summary of what was done and what was found | 3 | Individuals with inflammatory bowel disease (IBD) are at higher risk for mental health disorders, including depression. However, literature examining severe outcomes of depression in IBD, including depression-related hospitalizations and suicide death, is limited. |
| Introduction | | | |  |
| Background/rationale | 2 | Explain the scientific background and rationale for the investigation being reported | 4 | This susceptibility to depression is partly due to the chronic, episodic nature of IBD that often demands major shifts in coping strategies and mindset. Furthermore, research has established the relationship between depression and IBD to be bidirectional. |
| Objectives | 3 | State specific objectives, including any prespecified hypotheses | 4/5 | Consequently, this study aimed to i. calculate the 1-, 3-, and 5-year cumulative incidence risk of depression-related hospitalizations and ii. calculate temporal trends of depression-related hospitalization and suicide in individuals with IBD. |
| Methods | | | |  |
| Study design | 4 | Present key elements of study design early in the paper | 5 | This retrospective study used administrative data to identify all individuals with IBD and outcomes of depression-related hospitalization and suicide death. |
| Setting | 5 | Describe the setting, locations, and relevant dates, including periods of recruitment, exposure, follow-up, and data collection | 5 | Identification of the IBD prevalent cohort from healthcare-based administrative data requires the use of DAD, NACRS/AACRS, and Practitioner Claims. The resulting prevalent IBD cohort spans fiscal year (FY) (i.e., April 1 to March 31 [e.g., FY 2005 = April 1, 2005, to March 31, 2006]) 2005/06 to 2021/22. |
| Participants | 6 | (*a*) *Cohort study*—Give the eligibility criteria, and the sources and methods of selection of participants. Describe methods of follow-up  *Case-control study*—Give the eligibility criteria, and the sources and methods of case ascertainment and control selection. Give the rationale for the choice of cases and controls  *Cross-sectional study*—Give the eligibility criteria, and the sources and methods of selection of participants | 5 | Individuals in these databases are identified with the Provincial Registry via the Alberta Health Care Insurance Plan (AHCIP), the latter of which captures approximately 99% of the Albertan population. |
|  |  | (*b*) *Cohort study*—For matched studies, give matching criteria and number of exposed and unexposed  *Case-control study*—For matched studies, give matching criteria and the number of controls per case | 6 | To assess the comparison of IBD cases to a non-IBD population, controls (those without IBD) were 10 to 1 age- and sex-matched to each IBD case. Controls were only included if they were active (alive) when their matched case was diagnosed with IBD. |
| Variables | 7 | Clearly define all outcomes, exposures, predictors, potential confounders, and effect modifiers. Give diagnostic criteria, if applicable | 7 | The primary outcomes of interest were: i. hospitalization for depression after a diagnosis of IBD and ii. suicide death. |
| Data sources/ measurement | 8* | For each variable of interest, give sources of data and details of methods of assessment (measurement). Describe comparability of assessment methods if there is more than one group | 7 | The covariates of interest included age, sex, IBD type, and geography. Data on age, sex, and geographical location were collected from the Provincial Registry database. |
| Bias | 9 | Describe any efforts to address potential sources of bias | 6 | A validated algorithm is then applied to mitigate possible misclassification bias to identify prevalent IBD cases. |
| Study size | 10 | Explain how the study size was arrived at | 6/7 | A validated algorithm is then applied to mitigate possible misclassification bias to identify prevalent IBD cases. After the prevalent IBD cohort was identified, an eight-year washout period was applied to identify all adult (≥18 years) incident cases, while a three-year washout period was applied to identify all pediatric (<18 years) incident cases. The resulting inception cohort was from FY 2010/11 to 2021/2022. |

Continued on next page

| Quantitative variables | 11 | Explain how quantitative variables were handled in the analyses. If applicable, describe which groupings were chosen and why | 8 | AAPCs were stratified by age, sex, and geographic location in both cases and controls, with cases further stratified by IBD type. |
| --- | --- | --- | --- | --- |
| Statistical methods | 12 | (*a*) Describe all statistical methods, including those used to control for confounding | 8 | Test of proportion was performed for depression hospitalizations and suicide deaths across all available stratifications (i.e., males vs. females).  Using the inception cohort, the 1-, 3-, and 5-year cumulative incidence risk with 95% confidence intervals (CI) of depression-related hospitalization were calculated for IBD cases.  Using the overall prevalent cohort, average annual percent change (AAPC) with 95% CI were calculated for depression hospitalizations using Poisson regression or negative binomial depending on data dispersion from FY 2005/06 to FY 2021/22. AAPCs with 95% CI were calculated for suicide deaths using log-binomial regression from FY 2005/06 to 2021/22. |
|  |  | (*b*) Describe any methods used to examine subgroups and interactions | 8 | AAPCs were stratified by age, sex, and geographic location in both cases and controls, with cases further stratified by IBD type. |
|  |  | (*c*) Explain how missing data were addressed | n/a | No missing data. |
|  |  | (*d*) *Cohort study*—If applicable, explain how loss to follow-up was addressed  *Case-control study*—If applicable, explain how matching of cases and controls was addressed  *Cross-sectional study*—If applicable, describe analytical methods taking account of sampling strategy | 8 | The number of incident depression-related hospitalizations over 1, 3 and 5 years served as the numerator, with the inception cohort for each year used as the denominator. Follow-up times were calculated from the diagnosis date of each IBD case. To ensure complete follow-up, individuals with less than one, three, and five years of follow-up (based on any potential registration end date) were excluded, respectively. |
|  |  | (*e*) Describe any sensitivity analyses | 8 | A sensitivity analysis was conducted to determine the AAPC of depression-related hospitalizations for IBD cases from FY 2005/06 to 2021/22 when cases were limited to one hospitalization per FY, given the possibility of multiple hospitalizations for one individual. |
| Results | | | | |
| Participants | 13* | (a) Report numbers of individuals at each stage of study—eg numbers potentially eligible, examined for eligibility, confirmed eligible, included in the study, completing follow-up, and analysed | 9 | Table 1 describes the distribution of demographic and depression-related hospitalizations among both IBD cases (n = 36,942) and controls (n = 264,085). |
|  |  | (b) Give reasons for non-participation at each stage | n/a | n/a |
|  |  | (c) Consider use of a flow diagram | n/a | n/a |
| Descriptive data | 14* | (a) Give characteristics of study participants (eg demographic, clinical, social) and information on exposures and potential confounders | 10 | Table 1. Case and control demographics for depression hospitalizations  Table 2. Case and control demographics for suicide death |
|  |  | (b) Indicate number of participants with missing data for each variable of interest | n/a | n/a |
|  |  | (c) *Cohort study*—Summarise follow-up time (eg, average and total amount) |  | The resulting prevalent IBD cohort spans fiscal year (FY) (i.e., April 1 to March 31 [e.g., FY 2005 = April 1, 2005, to March 31, 2006]) 2005/06 to 2021/22.  The resulting inception cohort was from FY 2010/11 to 2021/2022. |
| Outcome data | 15* | *Cohort study*—Report numbers of outcome events or summary measures over time | 9 | Table 2 describes the distribution of demographic and suicide deaths for both IBD cases (n = 99) and controls (n = 565). There were 95 suicide deaths in IBD cases spanning FY 2005/06 to 2021/22 (0.26%). |
|  |  | *Case-control study—*Report numbers in each exposure category, or summary measures of exposure |  |  |
|  |  | *Cross-sectional study—*Report numbers of outcome events or summary measures |  |  |
| Main results | 16 | (*a*) Give unadjusted estimates and, if applicable, confounder-adjusted estimates and their precision (eg, 95% confidence interval). Make clear which confounders were adjusted for and why they were included | 9 | The 1-, 3-, and 5-year cumulative incidences of depression-related hospitalization in those with IBD was 0.11% (95% CI: 0.07%, 0.18%), 0.29% (95% CI: 0.20%, 0.40%), and 0.56% (95% CI: 0.42%, 0.72%), respectively.  Hospitalizations for depression decreased with an AAPC of −3.38% (95% CI: −4.72, −2.03), from 2.55 (95% CI: 1.73, 3.37) per 1,000 patients with IBD in FY 2005/06 to 2.08 (95% CI: 1.61, 2.55) per 1,000 in 2021/22.  Suicide deaths in those with IBD remained stable (AAPC: −1.35%; 95% CI: −5.58, 3.06). |
|  |  | (*b*) Report category boundaries when continuous variables were categorized | 7 | Age at time of event (depression hospitalization or suicide death) is stratified as pediatrics (<18 years of age) early adults (≥18-40), middle adults (41-64), and elderly (≥65) for depression hospitalizations; and stratified as those <65 years of age and those ≥65 for suicide death. |
|  |  | (*c*) If relevant, consider translating estimates of relative risk into absolute risk for a meaningful time period | n/a | n/a |

Continued on next page

| Other analyses | 17 | Report other analyses done—eg analyses of subgroups and interactions, and sensitivity analyses | 11 | Hospitalization rates for depression decreased in those with Crohn’s disease and those with ulcerative colitis, with AAPCs of −4.78% (95% CI: −6.53, −3.00) and −1.71% (95% CI: −4.46, 1.11), respectively, with the former being significant (Supplemental Figure 1). Similarly, hospitalization rates for depression significantly decreased in both females and males, with an AAPC of −2.93% (95% CI: −4.61, −1.21) and an AAPC of −3.00% (95% CI: −6.15, −1.81), respectively (Supplemental Figure 2). Likewise, hospitalization rates for depression significantly decreased in early adults with an AAPC of −3.16% (95% CI: −5.65, −0.61), middle adults with an AAPC of −2.95% (95% CI: −4.80, −1.07), and elderly adults with an AAPC of −5.43% (95% CI: −8.60, −2.15); however, hospitalization rates for depression remained stable in pediatrics with an AAPC of 0.74%, though this AAPC was not found to be statistically significant (95% CI: −8.43, 10.85) (Supplemental Figure 4). Finally, hospitalization rates for depression significantly decreased in both urban and rural IBD cases, with an AAPC of −1.72% (95% CI: −3.40, −0.01) and an AAPC of −6.38% (95% CI: −8.60, −4.11), respectively (Supplemental Figure 4).  Sensitivity analysis of depression hospitalizations from FY 2005/06 to 2021/22 when cases were limited to one hospitalization per FY found the AAPC to be −4.56 (95% CI: −6.19, −2.90). |
| --- | --- | --- | --- | --- |
| Discussion | | | | |
| Key results | 18 | Summarise key results with reference to study objectives | 13 | This study represents the first population-based study to assess depression-related hospitalization and suicide in those with IBD, providing insight into the burden of severe mental health outcomes in a population highly susceptible to mood disorders such as depression. Depression is a major health concern with 0.56% of individuals with IBD hospitalized for depression within 5-years of diagnosis, and 0.25% dying by suicide over the 17-year study period. Importantly, from 2005 to 2021, depression-related hospitalizations in those with IBD decreased by 3.4% each year, following similar trends observed in the control population. |
| Limitations | 19 | Discuss limitations of the study, taking into account sources of potential bias or imprecision. Discuss both direction and magnitude of any potential bias | 16 | This study did not assess the relative risk of depression-related hospitalizations or quantify the differences between IBD cases and controls. Disease severity, which is not captured in administrative data, was not account for, nor were medications. The outcome of depression hospitalization was based on a validated approach using the primary diagnostic coding of the 25 available diagnoses, raising the possibility that some depression hospitalizations were missed when coded as a secondary diagnosis rather than the primary diagnosis. Finally, insufficient sample sizes did not permit the stratification of suicide data in the same manner as depression hospitalizations. |
| Interpretation | 20 | Give a cautious overall interpretation of results considering objectives, limitations, multiplicity of analyses, results from similar studies, and other relevant evidence | 16 | Despite decreasing trends in depression hospitalization, suicide deaths in those with IBD remained stable during the study period, indicating that advancements in recognizing and treating depression have not translated to this outcome. Considering this, IBD clinicians should prioritize increased screening for suicidal ideation in those with IBD. Future research should investigate the prevalence of suicidal ideation in individuals with IBD and assess whether early psychological intervention can help reduce suicide risk. |
| Generalisability | 21 | Discuss the generalisability (external validity) of the study results | n/a | n/a |
| Other information | |  | | |
| Funding | 22 | Give the source of funding and the role of the funders for the present study and, if applicable, for the original study on which the present article is based | 2 | Canadian Institutes of Health Research Project Scheme Grant (Reference number: PJT-197959). |

*Give information separately for cases and controls in case-control studies and, if applicable, for exposed and unexposed groups in cohort and cross-sectional studies.

**Note:** An Explanation and Elaboration article discusses each checklist item and gives methodological background and published examples of transparent reporting. The STROBE checklist is best used in conjunction with this article (freely available on the Web sites of PLoS Medicine at http://www.plosmedicine.org/, Annals of Internal Medicine at http://www.annals.org/, and Epidemiology at http://www.epidem.com/). Information on the STROBE Initiative is available at www.strobe-statement.org.
